# Effects of different ischemic preconditioning occlusion pressures on muscle damage induced by eccentric exercise: a study protocol for a randomized controlled placebo clinical trial

**DOI:** 10.1186/s13063-021-05285-7

**Published:** 2021-05-05

**Authors:** Eduardo Pizzo Junior, Allysiê Priscilla de Souza Cavina, Leonardo Kesrouani Lemos, Taíse Mendes Biral, Carlos Marcelo Pastre, Franciele Marques Vanderlei

**Affiliations:** 1 Post-Graduate Program in Physiotherapy at Universidade Estadual Paulista - FCT/UNESP, Presidente Prudente, SP Brazil; 2 Undergraduate Program in Physiotherapy and the Post-Graduate Program in Physiotherapy at Universidade Estadual Paulista - FCT/UNESP, Presidente Prudente, SP Brazil

**Keywords:** Ischemic preconditioning, Exercise, Musculoskeletal pain, Creatine kinase, Muscle fatigue, Stress, Physiological, Randomized controlled trial

## Abstract

**Introduction:**

Due to its greater generation of muscle strength and less metabolic demand, eccentric exercise has been widely used in rehabilitation and for improving physical fitness. However, eccentric exercise can induce muscle damage by providing structural changes and reduced muscle function, so even with the protection caused by the repeated bout effect from eccentric exercise, it is necessary to seek alternatives to reduce this damage caused by stress. Thus, ischemic preconditioning could represent an aid to reduce the damage muscle or increase the protective effect caused by eccentric exercise.

**Objectives:**

To compare the effects of ischemic preconditioning, using different occlusion pressures, on acute and delayed responses to perceptual outcomes, markers of muscle damage, and performance in post-eccentric exercise recovery.

**Methods:**

A randomized controlled placebo clinical trial will be carried out with 80 healthy men aged 18 to 35 years who will be randomly divided into four groups: ischemic preconditioning using total occlusion pressure, ischemic preconditoning with 40% more than total occlusion pressure, placebo (10 mmHg), and control. The ischemic preconditioning protocol will consist of four cycles of ischemia and reperfusion of five minutes each. All groups will perform an eccentric exercise protocol, and assessments will be carried out before, immediately after, and 24, 48, 72, and 96 h after the end of the eccentric exercise to evaluate creatine kinase, blood lactate, perception of recovery using the Likert scale, being sequentially evaluated, pain by the visual analog scale, pain threshold using a pressure algometer, muscle thickness by ultrasound, muscle tone, stiffness and elasticity by myotonometry, vectors of cell integrity through electrical bioimpedance, and maximal voluntary isometric contraction using the isokinetic dynamometer. The trial was registered at ClinicalTrials.gov (NCT04420819).

**Discussion:**

The present study aims to present an alternative technique to reduce muscle damage caused by eccentric exercise, which is easy to apply and low cost. If the benefits are proven, ischemic preconditioning could be used in any clinical practice that aims to minimize the damage caused by exercise, presenting an advance in the prescription of eccentric exercise and directly impacting on the results of post-exercise recovery.

**Trial registration:**

ClinicalTrials.gov NCT04420819. Registered on 19 May 2020; Last update 24 March 2021.

**Supplementary Information:**

The online version contains supplementary material available at 10.1186/s13063-021-05285-7.

## Introduction

Resistance training is the most commonly used training method for maintaining and improving strength and muscle mass [1], being characterized by concentric or eccentric muscle contractions [2, 3]. Evidence indicates that eccentric training programs have been widely used in musculoskeletal rehabilitation or for improving physical fitness [4, 5]. The lower metabolic demand makes eccentric exercise (EE) an alternative to obtain better hypertrophic adaptations when compared to concentric exercise, promoting a greater amount of strength [2, 6, 7].

Thus, this exercise presents an interesting alternative for clinical use and should be encouraged in the rehabilitation process, to improve performance and prevent injuries. However, it must be considered that EE produces marked acute responses, structural damage, and reduced muscle function [8]. Due to the high force exerted on these deformable units, deformation occurs, which causes muscle stiffness, and may influence the risk of injuries [9].

Exercise-induced muscle damage (EIMD) is characterized by morphological alterations, decreased performance with reduced range of motion and strength, edema, delayed-onset muscle pain, and muscle proteins in the blood, especially creatine kinase (CK).

The literature has pointed out that EE itself has a protective effect against muscle damage; however, there are also strategies to assist recovery, such as massage, photobiomodulation, cryotherapy, and compressive clothing [10–15]. Some pre-exercise strategies are also used as a way to minimize these damages, especially ischemic preconditioning (IPC) [16], which is characterized by the application of short periods of circulatory occlusion (ischemia) and reperfusion of a limb in the minutes or hours preceding the exercise, through insufflations and deflations of a pressure cuff [17, 18].

The IPC was first presented by Murry et al. [17] and applied to the musculoskeletal system by Takarada et al. [19] and consists of three to four cycles of ischemia and reperfusion with 5 min of ischemia and reperfusion varying from 3 to 5 min [17, 19]. Patterson et al. demonstrated that this strategy can be administered from one to two times a day, applying three to five cycles of ischemia and reperfusion, with 5 min of ischemia and reperfusion varying from 3 to 5 min [20].

This strategy has been considered attractive, because in addition to being low-cost and non-invasive, it has easy clinical applicability and provides an ergogenic aid to improve physical performance [18]. Recent studies have emphasized the effectiveness of IPC in assisting post-exercise recovery [21], which can reduce the damage caused by ischemia-reperfusion injury after EIMD. These ischemia-reperfusion injuries cause metabolic and contractile damage similar to those presented in the EIMD, thus causing an increase in the concentrations of intracellular calcium, and in the appearance of muscle proteins in the blood and cytokine markers such as lactate and CK [22, 23].

Thus, its application before exercise can reduce the magnitude of muscle damage and subsequent pre-inflammatory responses, directly stimulating the body’s physiological defense system and improving tissue integrity [16, 24]. Therefore, some form of post-exercise recovery measurement is required, which can be acquired through a Likert recovery perception scale that ranges from 0 to 10 [25].

However, studies have been trying to determine the best prescription parameters for this strategy. Cocking et al. [26] and Lindsay et al. [27] analyzed the IPC response dose. The first study [26] investigated whether different IPC protocols improve muscle endurance, with a four-cycle and an eight-cycle protocol. The results showed that the four-cycle protocol demonstrated an improvement in resistance performance compared to the eight-cycle protocol. The second study [27], on the other hand, examined whether IPC applied different numbers of times per day improves performance; however, daily frequency was not a determining factor for improving performance [26, 27]. Another factor that hypothetically can determine the results of IPC is the pressure of occlusion generated in the period of ischemia; however, no studies were found that analyzed these effects in the recovery period after an EIMD.

Based on the above, it is hypothesized that IPC will provide a protective effect against eccentric EIMD, reducing muscle pain and improving clinical indicators, without, however, impairing muscle function. In addition, these responses are expected to be more evident in the higher occlusion pressure since there is a greater increase in blood flow and high levels of post-ischemic adenosine. In addition, factors related to the IPC response dose may influence responses in post-exercise recovery. Therefore, the aim of the present study is to compare the effects of IPC, using different occlusion pressures, on acute and delayed responses to perceptual outcomes, markers of muscle damage, and performance in post-eccentric exercise recovery.

## Methods

### Study design

This is a single-blind, placebo-controlled, clinical trial, with four parallel groups conducted at the Center for Studies and Care in Physiotherapy and Rehabilitation at Universidade Estadual Paulista (FCT/UNESP), Presidente Prudente, SP, Brazil. The trial was registered at ClinicalTrials.gov (NCT04420819) and approved by the Research Ethics Committee of FCT/UNESP, Presidente Prudente, SP, Brazil (CAAE: 30765020.3.0000.5402). Informed consent form is available from the corresponding author on request.

On the consent form, participants will be asked if they agree to use of their data should they choose to withdraw from the trial. Participants will also be asked for permission for the research team to share relevant data with people from the Universities taking part in the research or from regulatory authorities, where relevant. This trial does involve collecting biological specimens for storage.

The study protocol follows the SPIRIT 2013 checklist (Standard Protocol Items: Recommendations for International Trials) [28] (Supplementary File 1) and the TIDieR (Template for Intervention Description and Replication) [29], so that the information and quality of reports of interventions are well described [30].

### Participants and population analysis

For this study, the sample will consist of 80 healthy male individuals, aged between 18 and 35 years. We chose to investigate only men due to the differences found in the level of muscle strength and power between the sexes [31, 32]. These participants will be recruited, will be recruited from a database of the Sports Physiotherapy Laboratory of FCT/UNESP and the community, through dissemination posters on the institution’s premises, social networks, and advertisements in the local media. These procedures are recommended by Treweek et al. [33] as strategies to improve participant recruitment.

Individuals who exhibit one or more of the following characteristics will not be included (1) the presence of any health condition that contraindicates or prevents EE; (2) diabetes and diagnosed arterial hypertension; (3) inflammatory, psychiatric, cardiovascular, and/or respiratory rheumatological disease; (4) being an alcoholic, using drugs, and/or being a smoker; (5) history of knee surgery (for example, meniscal repair and ligament reconstruction) or recent musculoskeletal injury to the lower limbs that may impair performance during tests or interventions (for example, muscle injury, tendinopathy, patellofemoral pain in the lower limbs, and/or back pain in the previous six months); (6) involvement in any type of training program during the study period; (7) engaging in a lower limb strength training program during the three months prior to participating in the study; (8) use of ergogenic supplements to improve physical performance and/or muscle mass and/or vasoactive drugs; and (9) having one or more risk factors predisposing to thromboembolism [34].

Participants will be excluded from the study if they: present any health problem that does not allow continuity, use medication, electrotherapy, or other therapeutic means that may interfere with any result, perform unusual or strenuous physical activities during the evaluation period, or wish to leave the study.

Participants will be instructed in advance not to perform any physical activity or to use any therapeutic form of pain relief or performance improvement during the data collection. In addition, the participant may experience pain, dizziness, and sweating common to the practice of exercise, if there are episodes of musculoskeletal injuries, individualized physiotherapeutic treatment will be offered.

**Fig. 1:**
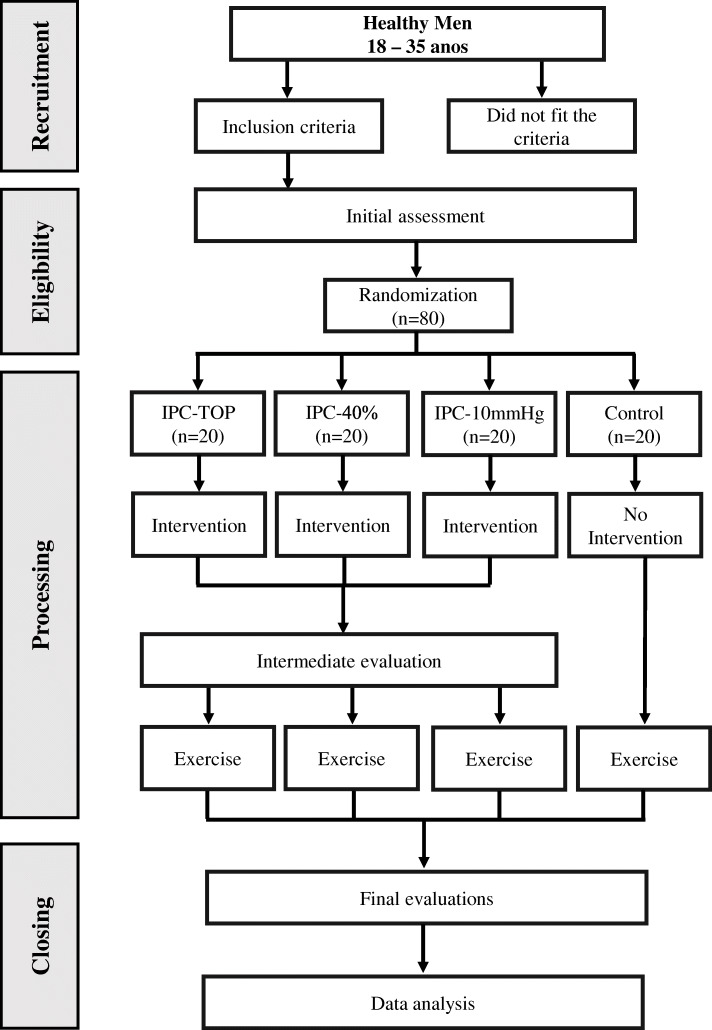
Flowchart of the study design. Legend: IPC-TOP (ischemic preconditioning with total occlusion pressure); IPC-40% (ischemic preconditioning with 40% higher pressure is to be applied than the closure pressure); IPC-10 mmHg (preconditioning with 10 mmHg)

The flowchart of the study design and the composition of the groups is illustrated in Fig. 1.

### Randomization

The randomization sequence will be developed by a researcher who will not be involved in recruiting, evaluating, or training participants using software (Microsoft Office Excel 2007) and will be placed in sequential numbering in opaque and sealed envelopes. There will be blinding of the evaluators and statistical analysis procedures. The experimental protocol will be supervised by trained physiotherapists who are not involved in the randomization process or assessments. Due to the nature of the interventions, the participants who perform the exercise will not be blind to the allocation of the groups. There is a Trial Steering Committee that meets weekly to provide daily support for the study. Any changes to the protocol will be notified to the ethics committee and an update will be made to the clinical trial record.

Participants who meet the eligibility criteria will be randomly allocated and balanced in a 1:1:1:1 ratio to one of the four groups, namely:

1. IPC-total occlusion pressure (TOP) [IPC-TOP]: this group will carry out baseline assessments, perform IPC using exactly the TOP, then perform post-IPC assessments and start EE. Post-exercise assessments will take place immediately after the end of EE and will be repeated at 24 h, 48 h, 72 h, and 96 h. All steps take place immediately after the end of the previous step, with the exception of the 24 h, 48 h, 72 h, and 96 h steps.
2. IPC-40%: this group will perform baseline assessments, perform IPC using 40% higher pressure is to be applied than the closure pressure, then perform post-IPC assessments and start EE. Post-exercise assessments will take place immediately after completion of the EE and will be repeated at 24 h, 48 h, 72 h, and 96 h. All steps take place immediately after the end of the previous step, with the exception of the 24 h, 48 h, 72 h, and 96 h steps.
3. IPC-10 mmHg: this group will carry out baseline assessments, perform the occlusion-perfusion intervention with 10 mmHg restriction characterizing the placebo, and then perform post-IPC assessments and start EE. Post-exercise assessments will take place immediately after completion of the EE and will be repeated at 24 h, 48 h, 72 h, and 96 h. All steps take place immediately after the end of the previous step, with the exception of the 24 h, 48 h 72 h, and 96 h steps.
4. CONTR: this group will carry out the baseline assessments, and immediately afterwards start the EE. Post-exercise assessments will take place immediately after the end of the EE and will be repeated at 24 h, 48 h, 72 h, and 96 h. All steps take place immediately after the end of the previous step, with the exception of the 24 h, 48 h, 72 h, and 96 h steps.

There will be no special criteria for discontinuing or modifying the allocated interventions.

### Study design

Data collection will be carried out at the Center for Studies and Assistance in Physiotherapy and Rehabilitation (CEAFIR) of FCT/UNESP, respecting the time from 17 to 22 h. All procedures will be performed under standard conditions (temperature: 21–23 °C; relative humidity: 40–60%). Each participant will attend the clinic for five consecutive days. Initially, participants will be assessed for anthropometric characteristics, using a scale (Tanita BC 554, Iron Man/Inner, Arlington Heights, IL, USA) and a stadiometer (Sany - American Medical do Brasil, São Paulo, Brazil) from which the body mass index (BMI) will be calculated.

**Fig. 2:**
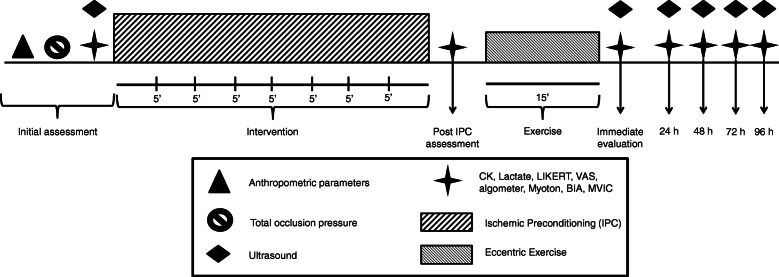
Study design

After these initial procedures, the TOP evaluation will be carried out. After a 10-minute rest, baseline outcome assessments will be performed. Initially, CK and blood lactate will be collected, followed by the application of the scales of perception of recovery and muscle pain and the pain threshold. In the supine position, the participant will rest for 10 min before the ultrasound, myotonometry, and bioelectrical impedance (BIA) evaluations are performed. Subsequently, MVIC will be evaluated. Next, the participants will perform the previously randomized 40-min IPC protocol. Evaluations will be carried out immediately after the IPC protocol of all analyzed outcomes except for ultrasound. The same order of execution of the evaluations will be maintained. Thereafter, EE will be initiated, and immediately after the end of the EE, all outcomes will be collected again. Subsequent visits will be carried out 24, 48, 72, and 96 h after the EE, in which the same outcomes mentioned above will be collected, maintaining the same order of execution of the evaluations. The study design is outlined in Fig. 2.

### Total occlusion pressure determination

After assessing the anthropometric parameters, the participants will be asked to lie down for 10 min [35]. All participants will be instructed to avoid strenuous exercise and alcohol intake within 48 h of the TOP assessment.

For the determination of TOP, a transducer with Doppler equipment (DV-2001; Medpej, Ribeirão Preto, São Paulo, Brazil) will be used, which will be positioned over the posterior tibial artery to capture the auscultatory pulse located at the average distance between the medial malleolus tibia and Achilles tendon. A blood pressure cuff will be attached to the participant's thigh close to the region of the inguinal crease of the dominant member [34] and will then be inflated to the point where the auscultatory pulse of the tibial artery is interrupted. TOP will be defined as the pressure at the moment when the arterial pulse is abolished, indicated by the absence of an auscultatory signal.

The vascular occlusion will be performed using an adapted blood pressure cuff (nylon, velcro, 175 mm wide and 920 mm long, JPJ - Hospital Materials Industry, São Paulo, Brazil). We opted for a wider cuff, as it has been proven that the width of the cuff has a great influence on the pressure required to achieve total blood flow restriction [36].

### Ischemic preconditioning protocol

**Fig. 3:**
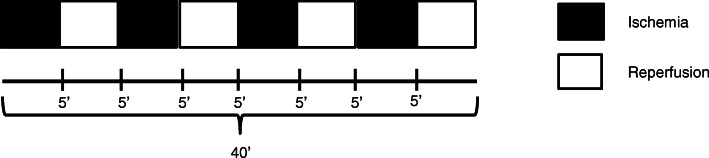
IPC protocol

The IPC protocol will be applied to the inguinal region of the dominant limb with the participants relaxed and comfortably positioned in the supine position. The same cuff used to determine TOP will be used and the protocol will consist of four cycles of total ischemia (TOP determined individually) of five minutes, followed immediately by four cycles of five minutes of vascular reperfusion (0 mmHg), totaling 40 min, as shown in Fig. 3.

To perform the IPC protocol, one of the study groups will use 40% more than the TOP, according to the study by Lopes et al. [37], the pressure at which the blood flow stops passing to the tibial artery varies from 140 to 160 mmHg and most studies performing IPC use pressures between 200 and 220 mmHg; thus, the values are corresponding [37]. The other study group will use the exact TOP, since at this value there is an absence of blood flow.

### Placebo preconditioning

Placebo preconditioning will be performed on the dominant thigh with the same cuff, similar to the IPC protocol described above (Fig. 3), but with four cycles of 5 min of placebo occlusion (10 mmHg), alternating with four cycles of 5 min of reperfusion (0 mmHg) [38, 39].

It should be mentioned that in the three study groups, participants will be previously informed that the applied occlusion pressure will be sufficient to improve performance and avoid muscle damage. In addition, all procedures will be performed individually, to prevent participants from talking to each other about the compression generated by the cuff.

### Eccentric exercise protocol

The EE will be performed on an isokinetic dynamometer (Biodex System 4 Pro, New York, NY, USA) for the knee extensor muscles of the dominant limb. Initially, five submaximal knee extension contractions will be performed for familiarization. Before each repetition, the dominant leg will be positioned at 30° of knee flexion. The participant will be instructed to perform a knee extension, while the dynamometer, with its resistance, returns the leg to 90° flexion, at a speed of 60°/s (1.04 rad/s) executing a range of movement of 60° (30–90° of knee flexion).

The protocol is based on the study by Machado et al. [25], starting 5 min after familiarization and consisting of 5 sets of 15 maximum eccentric contractions of knee extension, with 30 s of rest between sets, totaling 75 repetitions. The speed and range of movement will be similar to the familiarization and verbal encouragement will be given throughout the protocol. According to the authors, this protocol is capable of promoting muscle damage [25].

### Primary and secondary outcomes and assessment points

Primary outcomes will be related to muscle damage and muscle function through the quantification of creatine kinase, blood lactate, MVIC, and muscle thickness. In addition, there will be four measures of secondary outcomes: assessment of pain and pain threshold, assessment of recovery perception, myotonometry (muscle tone, stiffness, and elasticity), and electrical bioimpedance (resistance, reactance, and phase angle). All outcomes will be collected at the initial assessment with the exception of the perception of recovery, and immediately and 24, 48, 72, and 96 h after the end of the EE. Ultrasonography will not be performed immediately after the exercise.

### Details of procedures

#### Creatine kinase

Plasma CK concentration will be obtained by means of 32 μL of capillary blood collected from the digital pulp. This puncture will take place by means of a lancet with automatic trigger, after cleaning the location with 95% ethyl alcohol and drying with cotton. The blood sample will be drained into a heparinized capillary tube and then pipetted into a reactive CK strip for analysis on the Reflotron Plus System (Roche Diagnostics, Mannheim, Germany) using the reflection photometry method at 37 °C (test temperature). The test strips will be kept at a storage temperature of 2 to 8 °C, according to the manufacturer's instructions. The Reflotron System method allows fast and reliable measurement of CK levels [40].

#### Blood lactate concentration

To assess the participant’s blood lactate concentration, 25 mL of blood will be collected from an ear lobe capillary. Heparinized capillaries and polyethylene Eppendorf tubes (1.5 mL) containing 50 μL of sodium fluoride (NaF - 1%) will be used. The analyses will be performed on a lactimeter (YSI, Yellow Springs - 1500) [41] and the lactate values will be expressed in mmol/L. The lactate will be collected at the following moments: pre IPC protocol, pre EE, immediately after the end of EE, and in the 1st, 3rd, 5th, 7th, 9th, 11th, 13th, and 15th min, and 24 h, 48 h, 72 h, and 96 h after the intervention. The samples will be stored in a refrigerator with a temperature of 1 °C to 7 °C until their analysis.

#### Perception of recovery

The perception of recovery of the lower limb submitted to the EE protocol will be assessed using a 10-point Likert scale, where 1 indicates “not recovered” and 10 “fully recovered” [25]. The scale will be presented to the participants and so that they are not influenced by the researcher, they will answer the question: “From 1 to 10 points, how do you rate the perception of recovery felt in your lower limb at this moment?” [25, 42]. This scale will be applied immediately after the end of the EE, and 24, 48, 72, and 96 h after the intervention.

#### Muscle pain and pain threshold

Muscle pain will be measured using the visual analog scale (VAS). Participants will be asked to rate their exercise-induced leg pain, on the scale, which ranges from 0 “no pain” to 10 “extreme pain” [43].

To assess the pain threshold, a pressure algometer will be used, which is a reliable and validated instrument (FPX 50/220; Wagner instruments, Greenwich, CT, USA) [44]. The pressure algometer will be applied 4 cm above the base of the patella, 15 cm below the antero-superior iliac spine (ASIS), midpoint between the patella and the antero-inferior iliac spine (AIIS), and 2 cm medial and 2 cm lateral in relation to the midpoint. The pain threshold will be defined in kgf and will not exceed 2.55 kgf, as suggested by Jӧnhagen et al. [45].

#### Muscle thickness

The evaluation of the muscular structure will be carried out using ultrasound images of the participant’s dominant lower limb, which will be captured using Siemens Sonoline Sienna equipment (Issaquah, WA, USA), together with a linear matrix transducer (48 mm, 7, 5 MHz) to determine the thickness of the rectus femoris (RF) and vastus lateralis (VL) muscles. Anatomical reference points and skin marks will be drawn on transparent sheets to ensure similar positioning of the ultrasound transducer in the same location in the evaluations.

The images will be taken between the midpoint of the greater trochanter and the lateral condyle of the femur [46]. The ultrasound transducer will be covered with water-soluble transmission gel and positioned perpendicular to the skin over the RF and VL and oriented parallel to the muscle fascicles [46]. The alignment of the transducer will be considered adequate when several fascicles can be drawn without interruption through the image [46].

#### Myotonometry

MyotonPRO (MyotonAS, Tallinn, Estonia) will be used to measure muscle tone, stiffness, and elasticity of the quadriceps femoris muscle [47]. The device will be positioned at 2/3 between the ASIS and the upper pole of the patella [48], at the midpoint between them and 2 cm to the medial and 2 cm to the side, 15 cm from the AIIS and 4 cm from the base of the patella.

The device will be positioned perpendicular to the evaluated region, with light pressure. This preload is controlled and corresponds to 0.18 N of initial compression of the subcutaneous tissue, after which an additional impulse of 0.40 N will be released, with a duration of 15 ms. This impulse will induce oscillation in the tissue, which will be damped or deteriorated [49].

#### Electrical bioimpedance (BIA)

Electrical bioimpedance will be assessed using tetrapolar electrodes (BIA Analyzer, Nutritional Solutions Corporation, Harrisville, MI, USA, *f* = 50 kHz, and 800 μA) [50, 51]. Global and localized evaluations will be carried out. The global assessment will take place with the participant in the supine position; the electrodes will be positioned on the dominant hand (base of the 3rd metacarpal phalanx and between the styloid process of the radius and the head of the ulna) and on the dominant foot (at the base of the 3rd metacarpal phalanx and in the anterior region of the ankle, between the malleoli). The localized evaluation will be carried out to evaluate the quadriceps femoris muscles; the electrodes will be positioned five centimeters below the EIAS and above the base of the patella. The BIA components analyzed will be resistance R, reactance (Xc), and phase angle (phA) [51, 52]. The analysis of the tolerance ellipse will also be performed. The Bioscan program: BL-960141 (Biologica, Barcelona, ​Spain) will be used for the analyses [50, 51].

#### Maximal voluntary isometric contraction (MVIC)

For MVIC evaluation, the participant will be positioned with the dominant lower limb on the Biodex System Pro isokinetic dynamometer (Biodex Medical System, Shirley – NY, USA). According to the protocol suggested by Baroni et al. [53], prior to the evaluation, the volunteer will be submitted to a warm-up, which will consist of ten repetitions of concentric contractions of knee flexion-extension at 180°/s throughout the range of motion.

Muscle function will be assessed by means of the highest torque value obtained between three repetitions of 5 s of maximum voluntary contraction at 60° of knee flexion (with 0° corresponding to the maximum extension). A two-minute interval between repetitions will be administered in order to minimize the possible effects of fatigue. The participant will be instructed to give their maximum strength and will be verbally encouraged by the researcher in each maximum voluntary contraction.

#### Sample calculation

The sample calculation was performed based on the study by Bailey et al. [54], using the CK variable, since this variable expresses the amount of muscle protein in the blood representative of indirect muscle damage. The G*Power 3.1.9.7 software was used. Considering the standard deviation in the CK concentration of 200 μL, using a two-tailed hypothesis test with 80% test power and 5% significance level, a sample size of 16 participants per group was stipulated. Predicting sample loss, 4 participants per group were added totaling 80 participants. To increase the participants’ adherence to the intervention, telephone contact will be made daily to encourage them to remain in the study, and thus avoid losses. In case of discontinuity or withdrawal, the intention to treat will be used.

### Statistical analysis

The normality of the data will be verified by the Kolmogorov Smirnov test. If normality is detected, the sample characterization variables will be presented as mean and standard deviation, if not, as median and interquartile range. To compare the characterization of the sample, one-way ANOVA with Tukey’s post test or Kruskal-Wallis with Dunn’s post test will be used depending on the normality of the data.

The comparisons of the outcomes between the four groups studied and the moments will be performed using the technique of analysis of variance for repeated measures model in the two-factor scheme, which will provide information on the effects of time, group, and interaction. The repeated measurement data will be checked for sphericity violation using Mauchly’s test and the Greenhouse-Geisser correction will be used when the sphericity is violated. For moment analysis, Bonferroni’s post-test for parametric distribution or Dunn’s post test for non-parametric distribution will be used and the analysis between the groups will be performed using one-way ANOVA or the Kruskal-Wallis test.

In addition, the effects of the groups studied will be verified for all the outcomes assessed by calculating the effect size (ES) using Cohen’s *d*, considered as “null” (< 0.2), “small” (≥ 0.2), “moderate” (≥ 0.6), “large” (≥ 1.2), or “very large” (≥ 2.0) [55].

An intention-to-treat analysis will be performed using the patient’s most recent assessment in case of withdrawals or absence of data. The level of significance will be p < 0.05 for all tests. The statistical program SPSS (version 24.0) (SPSS Inc., Chicago, IL, USA) will be used for the analyses.

The external evaluator will enter the data into the database for screening, randomization, and statistical analysis purposes. Double data entry in electronic format will be used. Data integrity will be monitored by regularly scrutinizing data files for omissions and errors. Participants will be given an anonymous study ID to protect confidentiality, and only study investigators will have access to the final trial data set. The spreadsheets containing the raw numeric data of the data generated in this study will be stored in its entirety initially on two external hard drives and two online clouds. Any data required to support the protocol can be supplied on request.

## Discussion

### Potential impact and significance of the study

This IPC protocol follows the application time pattern found in the literature, however, with different occlusion pressures. Thus, the study findings could bring a new strategy to reduce muscle damage caused by exercise, based on the dose response. Thus, if the hypothesis of the present study is proven, physically active people could benefit from the technique, since it is easily accessible and applicable, in addition to being low cost.

It is also worth noting that the study aims to analyze the participants’ perceptions of the different IPC protocols, especially pain and recovery. Thus, these results may add elements that favor the use of IPC protocols and guarantee the adherence of this pre-exercise strategy.

### Strengths and weaknesses of the study

A strong point of the study is the comparison between two different IPC protocols with a placebo group and a control group, thus making it possible to observe the true results found from each intervention. The fact that the technique uses only a pressure cuff makes it easy to apply and access, so that it can be implemented in prevention and rehabilitation centers. Another positive point of the study is the monitoring of the effects of the technique for up to 96 h after the intervention, in addition to the high methodological quality of the study characterized by prospective registration, randomization, blinding, and intention to treat approach. However, a limitation of the study is the fact that the therapist and participants are not blind. Finally, some participants may be injured due to the strenuous protocol of eccentric exercise performed.

### Contribution and clinical applicability

As already described, the technique is easy to apply, low cost, and non-invasive. It can be applied in different environments of prevention, rehabilitation, and functional recovery. The data obtained in the present study could be used for better application of the technique in physiotherapy, especially in sports clinical practice, considering the periods of training and, especially, competition, providing protection from EIMD.

It is also worth noting that this study includes the items on the checklist for protocol studies in order to minimize bias, and was prospectively recorded. The outcomes will be disseminated through publications in scientific journals and presentations at congresses in the area.

### Trial status

Number Protocol: NCT04420819

Patient recruitment is currently underway.

Study start date: April 2021

Primary completion date: June 2021

Study completion date: December 2021

Finished recruitment: November 2021

## Supplementary Information

**Additional file 1.**

## Data Availability

The spreadsheets containing the raw numeric data will be stored on two external hard drives and two “clouds.” After analyzing the data, scientific articles related to the study will be made, and after publication, the data will remain preserved and will also be shared and made available in its entirety for a period of 5 years. The datasets analyzed during the current study are available from the corresponding author on reasonable request.

## Abbreviations

BIA: Electrical bioimpedance
CEAFIR: Center for Studies and Assistance in Physiotherapy and Rehabilitation
CK: Creatine kinase
MVIC: Maximal voluntary isometric contraction
EIMD: Exercise-induced muscle damage
EE: Eccentric exercise
ASIS: Antero-superior iliac spine
AIIS: Antero-inferior iliac spine
VAS: Visual analog scale
IPC: Ischemic preconditioning
TOP: Total occlusion pressure
RF: Femoral rectum
VL: Vastus lateral
